# What silent mutations say about the human airways

**DOI:** 10.7554/eLife.01541

**Published:** 2013-10-22

**Authors:** Matthew L Donne, Jason R Rock

**Affiliations:** 1 **Matthew L Donne** is at the Department of Anatomy, University of California, San Francisco, United States; 2 **Jason R Rock** is at the Department of Anatomy, University of California, San Francisco, United Statesjason.rock@ucsf.edu

**Keywords:** Human lineage tracing, mtDNA mutations, lung basal progenitor stem cells, stochastic homeostasis, airways, stem cells, Human

## Abstract

A technique for tracing stem cells and their descendants reveals how the lining of the airways is maintained, and how this process is altered in smokers.

**Related research article** Teixeira VH, Nadarajan P, Graham TA, Pipinikas CP, Brown JM, Falzon M, Nye E, Poulsom R, Lawrence D, Wright NA, McDonald S, Giangreco A, Simons BD, Janes SM. 2013. Stochastic homeostasis in human airway epithelium is achieved by neutral competition of basal cell progenitors. *eLife* **2**:e00966. doi: 10.7554/eLife.00966

**Image** Lung cells descended from a common progenitor can be identified on the basis of whether they express (green) or do not express (red) the mitochondrial gene *CCO*

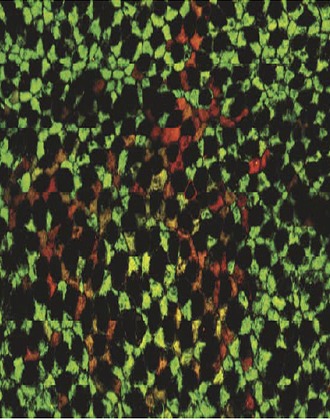

The lungs contain several different regions, including a network of tubes that filter and transport gases, and structures called alveoli in which carbon dioxide gas is exchanged for oxygen. Each of these regions is lined with a layer of tissue called the epithelium, which is maintained by a population of cells known as progenitors that replace old and damaged cells (Rock and Hogan, 2011). Experiments in mice have identified a group of progenitors called basal cells, which can both self-renew (i.e., replicate to produce more basal cells) and give rise to the other cell types that line the tubes, including ciliated cells and secretory cells (Rock et al., 2009). Basal cells thus fulfill the criteria for adult stem cells.

However, a number of questions remain. Are there special subsets of basal cells, perhaps localized to particular regions of the lung or expressing a unique repertoire of genes, that are capable of long-term self-renewal and differentiation? Do human airway basal cells also exhibit these behaviours in vivo? And could changes in these behaviours contribute to airway pathology? Now, in *eLife*, Sam Janes of University College London and co-workers—including Vitor Hugo Teixeira as first author—address these questions by tracing, for the first time, individual stem cells and their descendants in the airway epithelium of healthy humans and smokers (Teixeira et al., 2013).

To map the fates of stem cells, Teixeira et al. used a technique known as clonal lineage analysis, which involves following a genetic mark in adult stem cells that is reliably transmitted to all daughter cells. Because targeted genetic manipulations are not feasible in humans, there are currently few reports of lineage analysis of human progenitor cells in vivo. Instead, our knowledge of how human tissues are maintained has been extrapolated from in vitro and animal models.

To obtain in vivo data, Janes, Teixeira and co-workers—who are based at various institutions in London and Cambridge—exploited the fact that cellular organelles called mitochondria contain their own DNA, which accumulates mutations in a gene called cytochrome C oxidase (*CCO*). These spontaneous mutations are rare and do not affect the survival of cells, but they do result in loss of *CCO* expression. Because these mutations are irreversible and heritable, it is possible to identify mutated cells (and any cells descended from them) by their lack of CCO.

By sequencing the mitochondrial genomes of epithelial cells from human airway tissue sections, Teixeira et al. confirmed that mutant cells found adjacent to one another are clonal (i.e., one is descended from the other). They found that most groups of related cells (clones) contain at least one basal cell. A few rare clones lack basal cells, which can be attributed to stem cells that have undergone terminal differentiation (i.e., cells that have become another cell type rather than remain as a stem cell). These data are consistent with the hypothesis that the population of basal cells contains multipotent progenitor cells of the airway epithelium.

Next, Teixeira et al. devised a method for staining and imaging whole-mount human airways—pieces of tissue that have not been cut into thin sections—to more precisely determine the identity and behaviours of clones. They set out to test a model in which a single progenitor population (i.e., basal cells) maintains the airway epithelium through a process called ‘neutral drift’ (Figure 1). According to this model, some clones expand by chance through symmetrical self-renewal (in which a stem cell divides to produce two daughter stem cells), and this is exactly balanced by the loss of other clones as a result of stem cells undergoing terminal differentiation. A similar process has been shown to occur in the mouse intestine, oesophagus and other tissues (Clayton et al., 2007; Snippert et al., 2010; Doupe et al., 2012). The data Teixeira et al. obtained for the human airways also fit this model.

**Figure 1.**
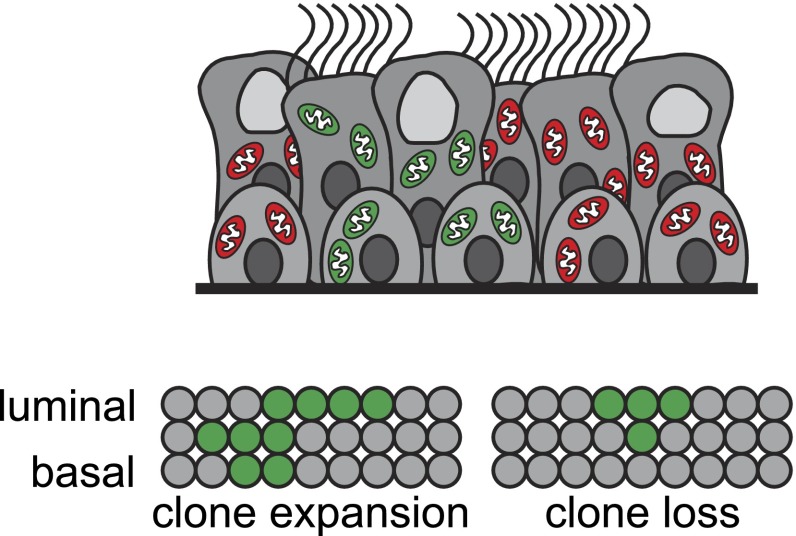
Stem cells known as basal cells maintain the lining of the airways. Genetic techniques can be used to identify groups of cells that are descended from the same progenitor (clones), represented by different colours in the figure (top). Teixeira et al. reveal that basal cells maintain the airway epithelium through a process called neutral drift. By chance, some clones expand via self-renewal (in which a stem cell divides symmetrically to produce two new stem cells) (bottom left); this is precisely balanced by the loss of other clones through terminal differentiation (in which stem cells commit to becoming other cell types) (bottom right). Each circle represents an individual cell.

Lastly, the researchers demonstrate that the expansion of clones to replace other cells—leading over time to the population of cells within the airway epithelium becoming increasingly uniform—occurs more rapidly in smokers than in non-smokers. At present, their data cannot determine whether this is due to an increase in the rate of proliferation, or to a shift in the ratio of symmetrical divisions (in which both daughter cells assume the same fate) vs asymmetrical divisions (in which one cell remains as a stem cell and the other differentiates). However, the data do establish the feasibility of analyzing the behaviour of cell clones in pathological contexts, including lung cancer.

By performing the first clonal analysis of human airways, this study has demonstrated that basal cells constitute a population of equipotent progenitors for the airway epithelium. Moving forward, it will be important to address the relative contributions of other progenitor populations, such as Clara cells, to airway maintenance and repair. Importantly, the model of neutral drift does not necessarily preclude a classical stem cell hierarchy in which a reserve of quiescent cells can be recruited to replace basal cell clones; similar hierarchies have been uncovered in other systems maintained by neutral drift (Mascre et al., 2012). Such airway epithelial cells would be of great importance for both basic and translational research.

The work of Janes, Teixeira and co-workers was not designed to determine the signals that regulate basal cell self-renewal and differentiation. It is possible that these processes are controlled by entirely intrinsic mechanisms, but it is equally possible that the loss and expansion of clones are coordinated through extrinsic mechanisms dependent on the local environment (stem cell niche). Understanding the signals that control these behaviours—including cell:cell interactions, cell:matrix interactions and secreted factors—will profoundly impact the development of cellular and molecular therapies for lung disease.
